# Association of birth weight with functional ovarian reserve during menacme estimated by serum concentration of anti-Müllerian hormone

**DOI:** 10.1038/s41598-019-44016-6

**Published:** 2019-05-30

**Authors:** Maria L. S. Lima, Gustavo S. Romão, Heloisa Bettiol, Marco Antonio Barbieri, Rui A. Ferriani, Paula A. Navarro

**Affiliations:** 1 Department of Gynecology, University of São Paulo, Ribeirão Preto (FMRP-USP), Ribeirão Preto, Brazil; 2 Department of Pediatrics, University of São Paulo, Ribeirão Preto (FMRP-USP), Ribeirão Preto, Brazil

**Keywords:** Gonadal disorders, Endocrine reproductive disorders

## Abstract

To investigate the relationship of birth weight (BW) of females born at full term with functional ovarian reserve (FOR) during menacme, based on serum level of anti-Müllerian hormone (AMH), among women who were 34–35 years old. This prospective birth cohort study assessed all women who were born in Ribeirão Preto City, State of São Paulo (Brazil) between June 1, 1978 and May 31, 1979. The primary endpoint was serum AMH, a marker of FOR, and its correlation with the BW of females classified as small for gestational age (SGA), appropriate for gestational age (AGA), and large for gestational (LGA). We included 274 women in this study, 19 were SGA, 238 were AGA, and 17 were LGA. The average of AMH concentration was not significantly different (*p* = 0.11) among women in the SGA group (2.14 ng/mL), AGA group (2.13 ng/mL), and LGA group (2.57 ng/mL). An analysis of variance indicated that the three groups also had no significant differences in the percentage of women who had adequate AMH levels (1 ng/mL; *p* = 0.11). There were no significant differences in the serum concentrations of AMH among 34 and 35 year-old women who were born at full term and classified as SGA, AGA, and LGA. Our sample size allowed detection of major differences between these groups (effect size of 0.8). Association of birth weight of females born at full term with functional ovarian reserve during menacme estimated by serum concentration of anti-Müllerian hormone.

## Introduction

A low functional ovarian reserve (FOR) is associated with advanced female age, a decline of natural fertility, and unsuccessful outcomes from assisted reproductive treatments (ARTs)^1^. The National Survey of Family Growth in United States concluded that the probability of not conceiving a first child within 12 months was less than 5% for 20 year-old women, but was almost 30% for 35 year-old women^2^. Moreover, a retrospective study of women receiving natural cycle single embryo transfer reported that the probability of obtaining a live birth was 26% for those under 35 years-old, but was 1% for those over 42 years-old^3^. This is a consequence of the significantly decreased FOR of women who are older than 40 years-old^1^.

Advanced female age is related to a reduced natural fertility and poor ART outcomes, and a high FOR is related to successful ART outcomes. Data on 145,660 ART cycles reported to the American Society of Reproductive Medicine in 2012 indicated that low FOR was responsible for about 16% of unsuccessful ARTs, and the rates of live births after ART were significantly lower in women with low FOR than those with male factor infertility, endometriosis, ovulatory disorders, and idiopathic dysfunction^4^. Moreover, about 41% of the women who had low FOR were less than 35 years-old^4^. Hence, identification of risk factors for low FOR at a young age is crucial for better child-birth counseling, especially because many couples delay fertility treatments for social, economic, or other reasons.

FOR also appears to be affected by epigenetic modifications of genes that control organs and organ systems, including the reproductive system. In particular, Barker’s hypothesis postulates that an infant’s birth weight (BW) is influenced by genetic factors inherited from the father and mother, and by the intrauterine environment, and that an adverse intrauterine environment alters the expression of certain genes that regulate the development and function of organs and tissues, and this ultimately increases the risk for some diseases^5^. In support of this hypothesis, some studies demonstrated that an unfavorable intrauterine environment may increase the risk of newborns being small for gestational age (SGA) due to the epigenetic reprogramming of fetal organs and tissues, leading to comorbidities during childhood^6^. For example, girls who were born SGA have a reduced ovulation rate during adolescence^7^ and a higher risk of polycystic ovary syndrome (PCOS)^8^. It is unknown whether the same genetic factors that lead to low BW also regulate FOR. However, an early age of menopause, which is associated with low FOR^9^, appears to be a complex phenotype that is influenced by multiple genetic factors^9^.

We hypothesized that an adverse intrauterine environment is reflected by low infant BW, and this can reprogram genes that regulate FOR. This hypothesis predicts a lower FOR (estimated by the serum level of anti-Müllerian hormone, AMH) in women who were born as SGA than in those who were large for gestational age (LGA). To test this hypothesis, we investigated the relationship of the BW of full-term females with FOR during menacme (estimated by serum AMH concentration) in women who were 34–35 years-old.

## Results

**Figure 1:**
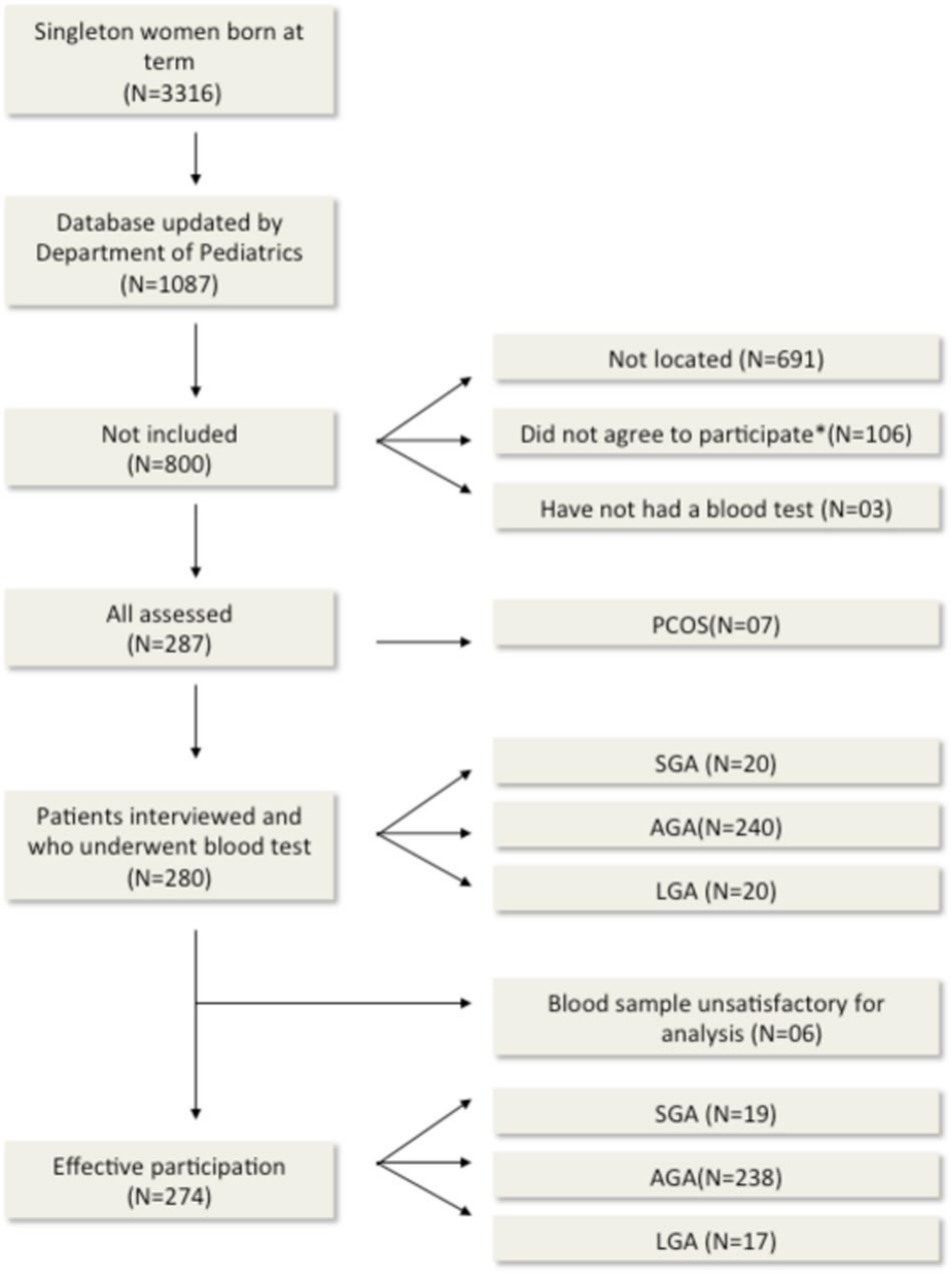
Flow chart of the study. Did not accept to participate: 83 patients refused to participate due to lack of time; 6 changed address and could not be located, 4 were breastfeeding; 9 were pregnant; 1 died; 1 performed recent bariatric surgery at the time of the call; 1 was in a vegetative state and 1 was deficient and could not leave home unattended. PCOS- Polycystic ovary syndrome. AGA, adequate for gestational age; SGA, small for gestational age; LGA, large for gestational age.

We identified 1087 women who were born at full-term from the updated database of Department of Gynecology and Pediatrics. We initially excluded 800 women: 691 who could not be located because of a change in address or a change in name after marriage, 106 who refused to participate, and 3 who did not provide blood samples (Fig. 1). Among the remaining 287 women assessed for eligibility, we excluded 7 women because they were diagnosed with PCOS in the 2007–2008 study^8^, in an effort to minimize confounding. Thus, for our analysis of 2012–2014, there were 280 eligible women who were born at full term, all between 33 and 34 years-old; 20 women were SGA, 240 were AGA, and 20 were LGA. After collection of blood samples, we excluded 6 women whose samples were not suitable for analysis. Thus, 274 women ultimately participated in this study, 19 were SGA, 238 were AGA, and 17 were LGA. The proportions of women who were SGA, AGA, and LGA in the original 1978/79 cohort were similar (Table 1).

**Table 1:** Percentage of SGA, AGA and LGA in the original cohort and current cohort.

Original 1978’s Cohort	2014–2015’s Cohort
10% SGA (318/3185)	7% SGA (19/274)
84% AGA (2683/3185)	87% AGA (238/274)
6% LGA (184/3185)	6% LGA (17/274)

Table 2 shows the social/ethnic characteristics, life habits, and clinical aspects of the volunteers included in the study. There was no significant difference between the three groups in relation to race, schooling, stable relationship, alcoholism, smoking, coffee intake, contraceptive method, previous gestation, previous chemotherapy, previous radiotherapy and previous ovarian surgery. As abortion was the only characteristic that indicated differences between the groups, with a trend towards a higher prevalence of abortions in SGA patients, a correlation analysis was performed and the Spearman coefficient was observed to be −0.176, pointing to a low correlation with a p value of 0.0026. In relation to the continuous variables, the only difference observed between the groups was weight (p < 0.01), with PIG infants reaching a final weight in adult life comparatively lower than the GIG infants (they obtained the highest weights of the sample), while AIG presented intermediate weights between these groups. An ANOVA test was performed, group by group, confirming this relation only in the comparison of the PIG group with AIG (p < 0.01) and PIG with GIG (p < 0.01), as shown in Table 3.

**Table 2:** Baseline Characteristics of the Patients.

Characteristic	SGA (n = 19)	AGA (n = 238)	LGA (n = 17)	Not reported	P value
Age (years)	34,13 ± 0,56	34,04 ± 0,57	33,82 ± 0,39	0 (0)	
Race no. (%)				1 (0)	0.43
White	14 (73.7)	198 (83.5)	16 (94.1)		
Non white	5 (26.3)	39 (16.4)	1 (5.9)		
Years of formal study no. (%)				6 (0)	0.84
≤8 years	2 (9.1)	33 (14.2)	1 (5.9)		
8–12 years	13 (68.4)	111 (47.8)	7 (41.2)		
≥12 years	5 (26.3)	88 (38.0)	9 (53.0)		
Alcoholism no. (%)	4 (21.0)	52 (21.0)	6 (35.3)	0 (0)	0.43
Smoker no. (%)	5 (26.3)	31 (13.0)	1 (5.9)	0 (0)	0.17
Stable relationship no. (%)	16 (72.7)	194 (80.8)	14 (82.4)	2 (0.0)	
Coffee intake no. (%)	15 (78.9)	168 (70.6)	15 (88.2)	0 (0)	0.23
Contraceptive use no. (%)	8 (42.1)	111 (46.8)	8 (41.7)	1 (0)	0.92
PCOS no. (%)	2 (10.5)	27 (11.3)	4 (23.5)	0 (0)	0.32
Parity no. (%)	14 (73.7)	159 (66.8)	11 (64.7)	0 (0)	0.81
Abortion no. (%)	6 (31.6)	29 (12.2)	0 (0)	0 (0)	**0.01**
Previous chemotherapy no. (%)	0 (0)	1 (0.4)	0 (0)	0 (0)	0.93
Previous radiotherapy no. (%)	0 (0)	1 (0.4)	0 (0)	0 (0)	0.93
Previous ovarian surgery no. (%)	0 (0)	3 (1.3)	0 (0)	0 (0)	0.79
Weight (kg)	63,4 ± 18,1	74,9 ± 17,12	80,6 ± 23,9	6 (0)	<**0.01**
Height (cm)	158,2 ± 6,6	155,6 ± 35	156,6 ± 41	5 (0)	0.95
Waist circumference (cm)	86.1 (77–100)	92.6 (84–102)	95.7 (85–101)	13 (0)	0.06
Number of living children	1 (0–2)	1 (0–2)	1 (0–2)	0 (0)	0.98

SGA, small for gestational age; AGA adequate for gestational age; LGA, large for gestational age; PCOS, polycystic ovary syndrome.

**Table 3:** Post-test group by group analyzing BW and weight reached in adult life.

Birth weight	p value
SGA × AGA	<0,01
SGA × LGA	<0,01
AGA × LGA	0,20

**Figure 2:**
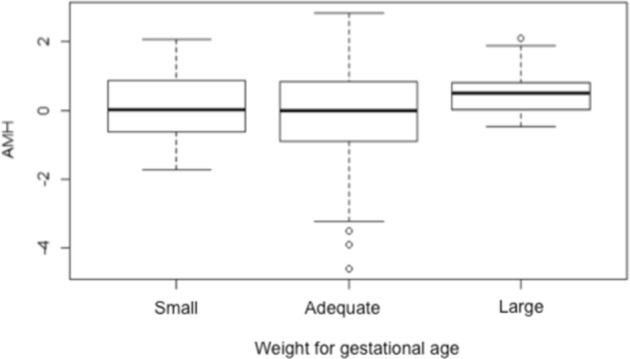
Boxplot of log_10_ AMH in relation to SGA, AGA and LGA groups.

The mean (±standard deviation) serum AMH level was 2.14 ng/mL (±2.46) in the SGA group, 2.13 ng/mL (±3.03) in the AGA group, and 2.57 ng/mL (±2.52) in the LGA group. After logarithmic transformation (because the distributions were skewed), an analysis of variance indicated no statistically significant differences among these groups (p = 0.11) (Fig. 2). According to Cohen^10^, an effect size of 0.80 is considered large. Thus, considering a power of 80% and our sample size (238 women with AGA [controls], 19 women with SGA, and 17 women with LGA), it was possible to discard a difference of 0.7 standard deviations in the SGA *vs*. AGA and LGA *vs*. AGA comparisons.

As a complementary analysis, we considered the AMH level as a categorical variable and considered an AMH level of 1 ng/mL and above as adequate, and a level below 1 ng/mL as inadequate (Table 4). Even with this categorization, there were no significant differences in the groups. In particular, 52.6% (10/19) of the SGA group had an adequate AMH and 47.4% (9/19) had a low AMH; 49.5% (118/238) of the AGA group had an adequate AMH and 50.5% (120/238) had a low AMH, and 76.4% (13/17) of the LGA group had an adequate AMH and 23.6% (4/17) had a low AMH (*p* = 0.09).

**Table 4:** Association between AMH and Birth weight.

AMH	Birth weight				P-value
	SGA (%)	AGA (%)	LGA (%)	Total (%)	
Adequate	10	118	13	141	0.09
	52.6	49.5	76.4	51.46	
Low	9	120	4	133	
	47.4	50.5	23.6	48.54	
Total	19	238	17	274	

AMH: Anti-mullerian hormone; P-value: Referring to Fisher exact test.

**Figure 3:**
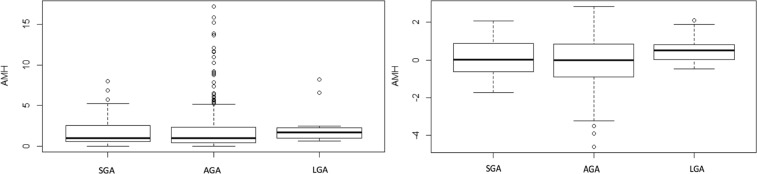
Boxplot for AMH distribution per group.

The observed distributions of AMH were skewed to the right for all groups, the log-AMH distribution on the other hand, did not show any skewness as showed in the Fig. 3.

## Discussion

The results of the present study indicated there were no significant differences in the serum concentrations of AMH among women who were 34 and 35 years-old who were born full-term and classified as SGA, AGA, and LGA. Moreover, there were also no differences among these groups in the percentage of women classified as having low or adequate FOR. Our sample size had a statistical power of 80%, so was suitable for identification of major differences among these groups.

Our results differ from those of two previous studies^11,12^ which reported that low BW adversely affected the FOR of women during their reproductive years. However, these studies were exploratory and based on indirect and imprecise markers of reproductive lifespan, FSH hypersecretion during childhood^11^ and ovary size reduction during adulthood^12^. We believe our results are more reliable because they are based on a direct and reliable marker of reproductive lifespan—serum AMH. In contrast, FSH elevation is a late predictor of menopausal transition, because it typically occurs about 10 years before menopause, and corresponds to the period when fertility has already declined^13^. Hence, a high level of FSH is not a reliable early predictor of fertility decline^14^. A decline in ovarian volume is also an imprecise predictor of menopause, because it has a high inter-cycle variation, so the result from a single month is not a reliable predictor of fertility.

The present study used the most reliable clinical marker of FOR—AMH^13^—and this is a major strength of our study. In addition, we evaluated a cohort of women who were treated at the Department of Child Care and Pediatrics of FMRP, and have reliable data on BW and GA at birth, because we obtained these data directly from the medical records of HCFMRP-USP (Clinical Hospital of FMRP). Another feature of our study that reduced bias is that all the women were born at full term; thus, GA at birth was not a variable. This classification considers the effect of intrauterine growth restriction, which has multiple causes. Knowledge of these parameters allows determination of the effects of fetal growth deficit while excluding the effect of prematurity^15^, which could be a confounding factor. Use of BW alone does not allow identification of premature babies, so this classification is inappropriate for study of the relationship between weight below p10 and the prevalence of diseases in adult life.

Studies in animal models^16^ have suggested that intrauterine malnutrition leads to follicular depletion and ovarian aging through oxidative stress^17^, and these may contribute to induction of follicular apoptosis^18^ and have adverse effects on oocyte maturation^19^. These studies also corroborate with Barker’s hypothesis, whose premise that impaired intrauterine growth and development may result in permanent changes in physiology and metabolism, thus increasing the risk of certain adverse events in adulthood such as coronary heart disease, hypertension, and type 2 diabetes^5,6,20^. These diseases may be consequences of “programming,” whereby a stimulus or insult at a critical, sensitive period of early life has permanent effects on structure, physiology, and metabolism. Although we are unaware of any previous studies that documented epigenetic effects of BW on subsequent FOR, some researchers have argued that some epigenetic factors can affect the age of menopause^21^, which could be considered a marker of FOR depletion. We suggest future studies perform more detailed investigations of the effect of epigenetic factors that affect both BW and FOR.

A limitation of the present study is the small number of cases in the SGA and LGA groups. This was due to the restrictive eligibility criteria and the difficulty in locating subjects after 34 years. However, as mentioned before, we intentionally used the same proportions of SGA, AGA, and LGA individuals as the original 1978/79 cohort. Moreover, our analysis allowed us to rule out large differences in the serum concentrations of AMH in the three study groups. Future studies using larger samples are needed to determine if there are small but statistically significant effects of BW on adequate FOR (AMH concentration of at least 1 ng/mL), especially by comparison of the LGA group with the AGA and SGA groups, using our data to guide sample size calculation. A second limitation is that it was impracticable to exclude all cases with PCOS because of the widespread use of hormonal contraceptives, which can influence AMH level^22,23^. But another study already showed that despite the association between oral contraceptive use and lower AMH levels, this same group of patients that were born SGA did not have its AMH levels affected^24^. Another limitation is that we lacked information about the previous pregnancies of the women’s mothers. Fetal growth restriction is a worst condition that happens when SGA is combined with abnormal cerebroplacental ratio, abnormal uterine artery or estimated fetal weight <3rd centile^25^. A study showed that miRNA expression is found in some pathological conditions as placental insufficiencies proving its association with the onset and progression of fetal growth restriction^26^. Without previous information about women’s mother pregnancies, it is hard to distinguish in the low birth weight group the woman who were SGA from those small for intrauterine pathological growth restriction, taking into consideration that certain comorbidities could also activate epigenetic effects that influence FOR. A fourth limitation is that there is no global standardization for measurement of serum AMH level^27^. This last limitation is a drawback for evaluations of this test, in terms of reproducibility and calibration (essential for the standardization of a single protocol), and for the performance of meta-analyses.

Our results are limited by the small sample size but the exclusion of large differences in AMH levels between birth weight groups is of paramount importance. Even though AMH is used as a tool to predict the response of ovarian stimulation, it is a poor predictor of pregnancy. AMH provides a quantitative evaluation but not a qualitative evaluation of ovarian reserve, which is provided essentially by age. In fact, AMH has a role in patient counseling and it is used in nomograms helping to choose the correct FSH starting dose in assisted reproductive technology treatments^28^ but it should not be used to predict inability to conceive, especially in women younger than 35 years old^29^. In this particular group of patients, the quality of oocytes and embryos is unaffected, even though the quantity of oocytes is diminished. Therefore, they have a much greater chance of pregnancy with their own eggs if they seek conception earlier than later^30^. That is why only a huge reduction of AMH could be considered of clinical relevance in woman aged 35 years old who may determine the consideration of these woman as at risk of premature ovarian insufficiency and this was excluded by this study.

It is not yet possible to state whether AMH level is a reliable predictor of reproductive success in women. It is important to standardize a protocol for measurement of AMH that has low intra- and inter-assay variability and low variability among different populations and ethnic groups, and to establish cut-off values and normal ranges for different age groups. Because of this, it is difficult to conclude whether a small difference in AMH level is clinically significant.

In conclusion, our results indicated that the BW of full-term females did not have a major impact on the FOR (estimated from serum AMH concentration) of Brazilian women who were 34 to 35 years-old. Nonetheless, it remains possible that BW could have a small or moderate effect on AMH level and FOR. Thus, further studies with larger sample sizes are needed.

## Methods

### Subjects

The study protocol was reviewed and approved by the Ethics Committee of Research of the Faculty of Medicine of Ribeirão Preto of the University of São Paulo (FMRP-USP) (number 5746/2012). This was a nested analytic study of a prospective birth cohort consisting of 6827 newborns (3316 females and 3511 males) from 1978 to 1979 in the city of Ribeirão Preto, State of São Paulo, Brazil. The 3316 females were assessed at 3 different times for other trials: twice by the Department of Pediatrics, FMRP-USP (1986–1987 and 2001–2002) and once by the Department of Gynecology (2007–2008). A total of 1087 females were born at full-term (gestational age of 37 to 42 weeks) and had updated medical records. We invited these women to participate in this survey from November 2012 to May 2014 using phone calls, e-mails, social networks, the University of São Paulo’s website, radio, television advertisements, and actively searching the municipal health registry. All patients provided written informed consent for participation. All participating women were born at full-term in Ribeirão Preto City between June 1, 1978 and May 31, 1979 and had reliable data on BW and gestational age (GA) at birth registered in their medical records. Women were excluded if they had amenorrhea with a hypothalamic-pituitary etiology, pregnancy, previous hysterectomy or bilateral oophorectomy, or a diagnosis of PCOS^31^ that was confirmed by participation in the previous 2007–2008 study^8^ by the Department of Gynecology. No cases of unilateral oophorectomy were found. Additional exclusion of other PCOS patients was not possible, because most of the women used hormonal contraception, precluding diagnostic confirmation by the Rotterdam criteria (2003)^31^.

### Clinical and laboratory evaluation

All clinical and laboratory evaluations were performed in the Laboratory of Gynecology of the University Hospital (FMRP-USP) during a single visit and after fasting for 12 h on any day of the menstrual cycle. Three researchers (MCB, MLSL, and OV) who were unaware of the neonatal data, performed all physical examinations and recorded demographic and clinical characteristics of the women. After a 20-min rest, venous blood was collected from each participant, centrifuged at 1600 g at room temperature for 10 min, and then stored at −70 °C, so that all samples were analyzed at the same time. The AMH concentration was determined using an immunoassay with the Ultra-sensitive AMH/MIS ELISA AL-105 apparatus (AnshLabs, Texas, USA®) through a quantitative immunoassay, based on the specific binding of the immunoglobulin to the antigen. All dosages were performed by the same observer, using the same kit, on the same day, blinded to which group the patients belonged. The assay was performed in 3 steps: first, the calibrators, controls and unknown samples were incubated with AMH antibody; in the second with biotinylated AMH antibody; and in the third, with a conjugated streptavidin solution (SHRP). The SHRP-antibody-antigen-biotin conjugate complex was detected by the enzyme-substrate reaction. The degree of enzymatic conversion of the substrate was determined by measuring the wavelength absorption at 450 nm and 630 nm. The measured absorption is directly proportional to the concentration of AMH in the samples and calibrators (kit ansh labs). The intra- and inter-assay coefficients of variation were 3.19 ng/mL ± 0.19 ng/mL and 1.74 ng/mL ± 0.16 ng/mL, respectively.

### Outcome measures

All women were placed into 3 groups according to the BW for full-term newborns using William’s criteria^32^: SGA, BW below the tenth percentile (p10); appropriate for gestational age (AGA), BW between p10 and p90; and LGA, BW above the tenths percentile (p90).

### Sample size calculation

Previous studies only examined correlations of BW with indirect markers of reproductive potential, such as follicle stimulating hormone (FSH)^11^ and ovarian volume^12^. The present study is unique because it uses AMH, a more accurate and reliable marker of FOR. Because our strict inclusion criteria would make it difficult to recruit a sufficient number of women, the sample size was calculated using the assumption of Cohen^10^, which considered an effect size of 0.80. Thus, for a power of 80%, it would be necessary to evaluate 20 SGA women, 240 AGA women, and 20 LGA women to discard, in the two comparisons SGA vs. AGA and LGA vs. AGA, a difference above 0.66 standard deviations (SD).

### Statistical analyses

AMH centrality and dispersion measures in the SGA, AGA, and LGA groups were compared and box-plots to show the distributions of AMH levels as a function of BW. Analysis of variance was used to compare the FOR of the three groups. Because the AMH level had a skewed distribution, it was log_10_-transformed before the analysis of variance. Statistical analyses were performed using SAS software, version 9.3 (SAS Institute Inc., University of North Carolina, USA) with the PROC GLM procedure. The level of significance for all analyzes was set at 5%. In a complementary analysis, AMH was categorized as adequate if it was 1 ng/mL or more and inadequate if it was below 1 ng/mL. This categorization process was based on previous studies using 1 ng/mL cut-off point or approximate values for subfertile women (Shebl, Ebner *et al*., 2011), increased chance of presenting poor response in cycles of ovarian stimulation (AMH < 0.99 ng/mL)^33^ or reduced chance of live birth (AMH < 1.05 ng/mL)^34^. Based on these data, this value of 1 ng/mL was chosen for categorization of AMH, being below 1 ng/mL considered low ROF and above 1 ng/mL adequate ROF. We then tested the null hypothesis that there are no differences in the proportions of women with adequate AMH in the three groups using Fisher’s exact test. For this last analysis, the PROC FREQ procedure in SAS was used.

### Compliance with ethical standards

Research involving Human Participants. All procedures performed in studies involving human participants were in accordance with the ethical standards of the institutional and/or national research committee and with the 1964 Helsinki declaration and its later amendments or comparable ethical standards.

## Data Availability

The datasets generated during and/or analysed during the current study are available in the figshare repository, 10.6084/m9.figshare.7460660.
